# Characterization of *Escherichia coli* from Edible Insect Species: Detection of Shiga Toxin-Producing Isolate

**DOI:** 10.3390/foods10112552

**Published:** 2021-10-22

**Authors:** Anja Müller, Diana Seinige, Nils T. Grabowski, Birte Ahlfeld, Min Yue, Corinna Kehrenberg

**Affiliations:** 1 Institute for Veterinary Food Science, Justus Liebig University Giessen, Frankfurter Str. 92, 35392 Giessen, Germany; anja.mueller@vetmed.uni-giessen.de; 2 Lower Saxony State Office for Consumer Protection and Food Safety, 26203 Wardenburg, Germany; diana.seinige@gmx.de; 3 Institute for Food Quality and Food Safety, University of Veterinary Medicine Hannover, Foundation, Bischofsholer Damm 15, 30173 Hannover, Germany; Nils.Grabowski@tiho-hannover.de (N.T.G.); birteahlfeld@web.de (B.A.); 4 Institute of Veterinary Sciences & Department of Veterinary Medicine, College of Animal Sciences, Zhejiang University, 866 Yuhangtang Road, Hangzhou 310058, China; myue@zju.edu.cn

**Keywords:** Shiga toxin, *Escherichia coli*, antimicrobial resistance, edible insects

## Abstract

Insects as novel foods are gaining popularity in Europe. Regulation (EU) 2015/2283 laid the framework for the application process to market food insects in member states, but potential hazards are still being evaluated. The aim of this study was to investigate samples of edible insect species for the presence of antimicrobial-resistant and Shiga toxin-producing *Escherichia coli* (STEC). Twenty-one *E. coli* isolates, recovered from samples of five different edible insect species, were subjected to antimicrobial susceptibility testing, PCR-based phylotyping, and macrorestriction analysis. The presence of genes associated with antimicrobial resistance or virulence, including *stx1*, *stx2,* and *eae*, was investigated by PCR. All isolates were subjected to genome sequencing, multilocus sequence typing, and serotype prediction. The isolates belonged either to phylogenetic group A, comprising mostly commensal *E. coli*, or group B1. One O178:H7 isolate, recovered from a *Zophobas atratus* sample, was identified as a STEC. A single isolate was resistant to tetracyclines and carried the *tet*(B) gene. Overall, this study shows that STEC can be present in edible insects, representing a potential health hazard. In contrast, the low resistance rate among the isolates indicates a low risk for the transmission of antimicrobial-resistant *E. coli* to consumers.

## 1. Introduction

Edible insects are popular foods in many parts of the world such as Asia and Africa. In Europe, insects are still rarely consumed but are gaining in popularity. They are of particular interest to consumers due to their nutritional value as well as aspects of sustainability, including a lower need for feed, water, and space, when compared to traditional livestock [1,2].

In the European Union (EU), edible insects are included in the novel food regulation, Regulation (EU) 2015/2283, which lays the legal framework for placing novel foods on the European market. However, specific EU regulations regarding the production of insects intended for human consumption and controls thereof are still lacking, and the legal situation remains complicated. Edible insects can be novel food, as established in Regulation (EU) 2015/2283, if a corresponding application is answered favorably by the authorities. To date, several applications for edible insect products have been submitted. The European Food Safety Authority has already published a scientific opinion regarding the safety of dried yellow mealworm (*Tenebrio molitor*), which concluded that they are safe for human consumption [3], an important first step towards an approval for this species to be placed on the European market. More recently, the European commission has released the Commission Implementing Regulation (EU) 2021/882, authorizing the placing on the market of dried yellow mealworms as of 1 June 2021. This was the first time an insect species was officially approved to be marketed as food within the EU.

Beyond general regulations applicable to all foodstuffs, some countries have issued national guidelines concerning edible insects, while other countries do not currently have any specific national framework in place [4]. In 2019, the working group on meat and poultry hygiene and specific issues relating to food of animal origin of the German national working group on consumer health protection (Arbeitsgruppe Fleischhygiene und fachspezifische Fragen von Lebensmitteln tierischer Herkunft der Länderarbeitsgemeinschaft Verbraucherschutz, AFFL) issued corresponding recommendations [5]. To date, potential food safety concerns associated with insect-based products are still being evaluated. In 2015, the European Food Safety Authority (EFSA) published a scientific opinion stating which hazards were expected to be largely comparable to those posed by other foods of animal origin, while also highlighting remaining uncertainties and the need for further research [1]. This includes a recommendation to monitor bacteria and gather data regarding antimicrobial resistance [1]. This is particularly important as high microbial loads have previously been reported in insect-based foods [6,7]. Consequently, the presence of antimicrobial-resistant isolates could result in significant exposure for consumers. There is very little information regarding the use of antimicrobials in the rearing of edible insects; it has been recommended only as a temporary measure in case of emergency [8]. In addition, negative side-effects of antibiotic treatment, such as a lower number of eggs produced and poorer development in some insect species, can discourage the use of antimicrobials [9]. Consequently, this may result in a lower selection pressure and a lower frequency of antimicrobial-resistant bacteria in insects when compared to vertebrate livestock. However, to date, very few studies have been published regarding the characteristics of bacterial strains present on edible insect species, including their antimicrobial susceptibility and the presence of virulence genes [10,11].

*Escherichia coli* and Shiga toxin-producing *E. coli* (STEC), in particular, are among the most important and most frequently reported food-borne pathogens [12]. In humans, STEC can cause severe diarrhea, hemorrhagic colitis, and hemolytic uremic syndrome, which may ultimately be fatal [13]. Besides STEC, ESBL-producing and other antimicrobial resistant *E. coli* present on food of animal origin are of concern as they can facilitate the spread of antimicrobial resistance to the consumer [14,15].

However, to date, few studies have examined the presence of STEC in edible insects [11], and virtually no data are available regarding the antimicrobial resistance status of *E. coli* isolated from edible insects. Such data are crucial to elucidate their potential role in the transmission of antimicrobial resistance via the food chain and other health hazards associated with the consumption of insects. To the best of our knowledge, this is the first study specifically investigating the phenotypic and genotypic antimicrobial susceptibility, the presence of certain virulence genes, as well as the genetic relatedness of *E. coli* isolates obtained from different species of edible insects.

## 2. Materials and Methods

### 2.1. Insect Samples

A total of 36 samples of edible insect species were available to be included in this study. They were collected and tested for the presence of *E. coli* between 2014 and 2016 and consisted of two subsets. One subset was derived from the project “ZooGlow” that dealt with legally and illegally introduced and commercialized foodstuffs inside and outside Germany. It contained ten samples of frozen silkworms (Lepidoptera: Bombycidae: *Bombyx mori*) imported from Vietnam and purchased at an Asian supermarket in Germany. Two additional samples were powdered yellow mealworm larvae (Coleoptera: Tenebrionidae: *Tenebrio molitor*) bought online from a German retailer.

The other subset was insects bought at a local pet store, i.e., Mediterranean crickets (Orthoptera: Gryllidae: *Gryllus bimaculatus*; n = 1), migratory locusts (Orthoptera: Acrididae: *Locusta migratoria*; n = 4), *T. molitor* (n = 1), and superworms (Coleoptera: Tenebrionidae: *Zophobas atratus*, previously known as “*Z. morio*”; n = 3). More specimens and other species were also analyzed but did not yield *E. coli*, e.g., Jamaican field crickets (Orthoptera: Gryllidae: *Gryllus assimilis*), house crickets (Orthoptera: Gryllidae: *Acheta domesticus*), and desert locusts (Orthoptera: Acrididae: *Schistocerca gregaria*), with five samples (n = 5) each. The animals obtained from the pet store were explicitly not intended as foodstuff. However, many entomophagy aficionados in Western countries repurpose them as they point out the freshness of the product, the better taste, and a higher degree of culinary diversity, i.e., more different dishes can be made from hygienically processed pet feed insects than from freeze–dried (and sometimes spiced) entire or ground food insects (Nils Th. Grabowski, personal communication, 2017). For this reason, a microbiological evaluation of edible insects sold as pet feed is important for risk assessment. They usually let the animals fast for one day, kill them, and cook them thoroughly before processing them further. Samples for this analysis were raw and killed by freezing.

### 2.2. Isolate Collection and Species Identification

While doing a classical microbiological analysis of the insect samples, *E. coli* strains were detected and isolated according to standard methods described in DIN EN ISO 16649-2. In brief, samples were subjected to an enrichment step in NaCl-peptone water for 24 h at 37 ± 0.5 °C, and the broth was subsequently streaked on TBX agar (Oxoid, Wesel, Germany) for the selective detection of *E. coli*.

The species of the isolates was confirmed using a MALDI-TOF biotyper (Bruker Daltonics, Bremen, Germany) and by species-specific PCR targeting the *gadA* gene [16]. Only one isolate per sample was included in further investigations.

### 2.3. Antimicrobial Susceptibility Testing

Antimicrobial susceptibility testing was performed and evaluated in accordance with CLSI standards [17,18] using commercially available Sensititre microtiter plates (EUVSEC layout; Trek Diagnostic Systems Ltd., East Grinstead, UK) containing the following antimicrobials and concentrations: ampicillin (1–64 µg/mL), azithromycin (2–64 µg/mL), cefotaxime (0.25–4 µg/mL), ceftazidime (0.5–8 µg/mL), chloramphenicol (8–128 µg/mL), ciprofloxacin (0.06–8 µg/mL), colistin (2–16 µg/mL), gentamicin (0.5–32 µg/mL), meropenem (0.06–16 µg/mL), nalidixic acid (4–128 µg/mL), sulfamethoxazole (8–1024 µg/mL), tetracycline (2–64 µg/mL), tigecycline (0.25–8 µg/mL), and trimethoprim (0.25–32 µg/mL). *E. coli* ATCC 25922 was used for quality control purposes.

### 2.4. Molecular Analyses

*E. coli* isolates were assigned to the four major phylogenetic groups based on PCR assays detecting the genes *chuA*, *yjaA,* and the DNA fragment TSPEC4.C2 [16]. Clonal relatedness of the isolates was investigated by *Xba*I macorestriction analyses and subsequent pulsed-field gel-electrophoresis according to the PulseNet Protocol for *Salmonella*, *Shigella*, *E. coli* O157, and other Shiga toxin-producing *E. coli* [19]. The results were analyzed using BioNumerics V. 7.6 (Applied Maths, Sint-Martens-Latem, Belgium) applying the Dice coefficient with 0.5% optimization and 1% position tolerance.

**Figure 1:**
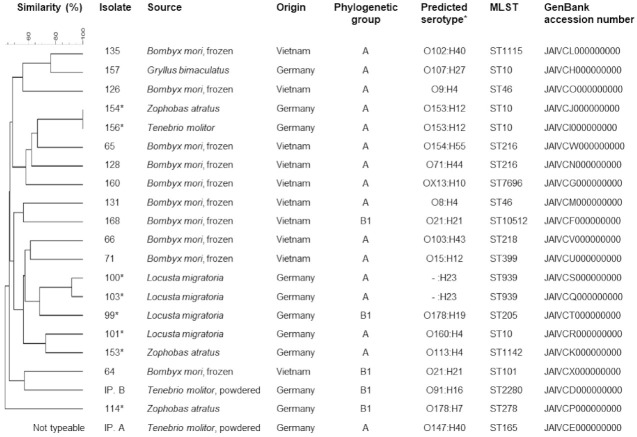
Origin and molecular typing results of the 21 *E. coli* isolates. Accession numbers refer to genome sequences deposited in the GenBank database. * Isolates recovered from samples purchased at pet feed store.

All isolates were subjected to full genome sequencing (MicrobesNG, University of Birmingham, UK) for further typing, including multilocus sequence typing and serotype prediction. Sequencing was performed on an Illumina sequencing platform using 2 × 250 bp paired-end reads. The following pipelines were used for the bioinformatics analyses included in their standard sequencing service: Kraken, BWA mem, SPAdes for de novo assembly of reads, and Prokka for automated annotation (see Figure S1 for assembly statistics). Genome sequences were deposited in the GenBank database (https://www.ncbi.nlm.nih.gov/genbank/ (accessed on 14 September 2021)), and accession numbers for all isolates are listed in Figure 1. The presence of antimicrobial resistance- and virulence-associated genes was investigated using ResFinder 4.1 [20] and Virulence Finder 2.0 [21], respectively. Genome sequences were additionally uploaded to the Enterobase *Escherichia/Shigella* database (http://enterobase.warwick.ac.uk/ (accessed on 13 September 2021)) for multilocus sequence typing (MLST) and serotype prediction. Assembly of Illumina reads within the Enterobase database was performed using QAssembly, and subsequently the prokka pipeline was run for annotation. A minimum spanning tree was created using the GrapeTree program within Enterobase, using the cgMLST V1 + HierCC V1 scheme and the NinjaJ algorithm [22].

## 3. Results

**Figure 2:**
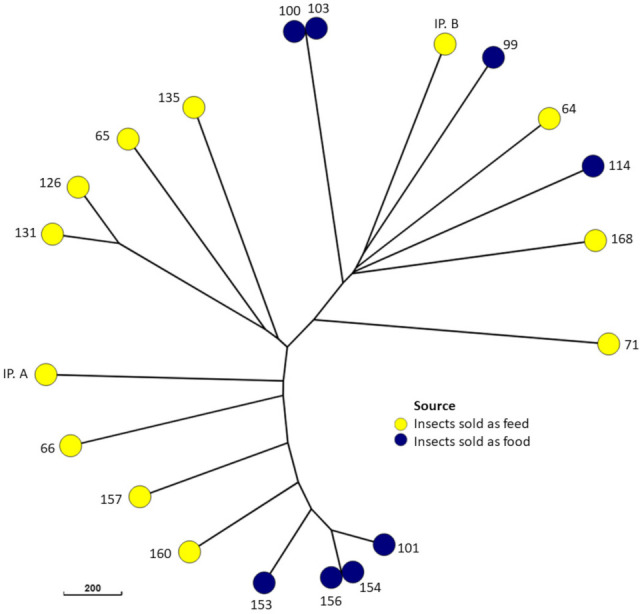
Minimum spanning tree of the 21 *E. coli* isolates based on core genome sequences.

A total of 21 *E. coli* isolates were recovered from the 36 samples. They were detected in five different insect species (Figure 1). No isolates were found in samples of Jamaican field crickets (*Gryllus assimilis*), house crickets (*Acheta domesticus*), or desert locusts (*Schistocerca gregaria*). The majority (n = 16) of the 21 isolates belonged to phylogenetic group A. The remaining isolates belonged to group B1 (Figure 1). PFGE analysis revealed identical band patterns of two isolates. These isolates were recovered from samples of superworms (*Zoophobas atratus*) and mealworms (*Tenebrio molitor*), respectively, which were both purchased from a German pet feed store. In addition, a clustering of the four isolates recovered from migratory locusts (*Locusta migratoria*) was observed, with the two most similar isolates showing a similarity of band patterns of over 90% (Figure 1). These clusters were also apparent in the minimum spanning tree created based on cgMLST (Figure 2). In addition, isolates 126 and 131, both recovered from *B. mori* samples from Vietnam, showed a comparatively close relatedness, which was not apparent in their respective macrorestriction patterns. The remaining isolates showed very heterogenous PFGE band patterns, with less than 45% similarity between the most dissimilar isolates. One isolate (IP. A) did not produce bands after *Xba*I digestion and was thus classified as not typeable by this method.

The most commonly detected multilocus sequence type was ST10 (n = 4, Figure 1). In one isolate of phylogenetic group B1, a novel *fumC* allele was identified, and it was assigned to a novel sequence type, designated ST10512, in the Enterobase database. Serotype prediction based on genome sequences revealed a variety of serotypes. Three serotypes were identified in two isolates each. The two ST10 isolates sharing identical PFGE band patterns both belonged to O153:H12, and two ST939 isolates, which also shared a high similarity of PFGE band patterns, shared the same H-type and lack of detectable O-antigen (-:H12). Two other isolates from silkworms belonged to serotype O21:H21 but showed dissimilar band patterns (Figure 1). Other serotypes were only identified in single isolates. One O178:H7 isolate was classified as a STEC, carrying genes for Shiga toxin types 1 and 2 (*stx1c*, *stx2b*). Based on genome sequence analyses, the nucleotide sequence of *stx1* subunit A and B, as well as *stx2* subunit B, showed 100% identity to reference sequences deposited in the GenBank database under accession numbers Z36901 (*stx1c*) and AF043627 (*stx2b*) [23]. In contrast, there were seven nucleotide differences in the sequence of *stx2* subunit A compared to that of the reference strain (for the gene *stx2b*; GenBank acc. no. AF043627). Three of these resulted in amino acid exchanges in the deduced protein, which were identical to the variant of Stx2b subunit A deposited under Genbank accession number WP_001367518. The isolate lacked intimin encoding gene *eae* but carried several other virulence genes, including enterohaemolysin gene *ehxA*, subtilase cytotoxin encoding gene *subA,* and adherence-associated gene *iha*, among others (Table 1). The remaining isolates carried few virulence genes overall. Besides *gad* and *terC*, which were present in all isolates, the increased serum survival gene *iss* was present in several isolates, and two isolates carried *astA*, encoding a heat-stable enterotoxin (see Table 1 for full virulence profiles).

Antimicrobial susceptibility testing revealed that only a single isolate showed resistance to tetracyclines. This isolate carried the *tet*(B) gene. The remaining isolates did not show resistance to any of the antimicrobial agents in our test panel. One isolate carried *qnrS2*, however, despite being in the susceptible range for both quinolones tested. The *mdf(A)* gene, encoding a multidrug efflux pump, was present in all isolates.

## 4. Discussion

The *E. coli* isolates included in this study were recovered from taxonomically diverse species and included holometabolous insects undergoing complete metamorphosis during development (Coleoptera, Lepidoptera), as well as hemimetabolous insects (Orthoptera). Previous research indicated that the microbiome of edible insects is usually dominated by Gram-positive bacteria, and in contrast to our current observations, *E. coli* were not typically detected [11,24]. Overall, *E. coli* does not appear to be a regular part of the microbiome of edible insects, and human pathogenic bacteria in general are likely obtained through feed, substrate, or during handling and processing [1]. While fecal bacteria in pet feed insects may be expected to some degree, the presence of *E. coli* in *B. mori* is more intriguing. They have been submitted to a heating process, and insect pupae should be sterile in the first place because larvae usually empty their intestinal tract content completely before pupation, and during it, the gut is dissolved and de novo synthesized. The larval gut turns into the so-called “yellow body” and is digested completely [25]. Thus, the presence of bacteria in silkworm pupa samples should be a result of secondary contamination during handling and processing. In fact, silkworm pupae from Vietnam were previously reported as positive for salmonellae via the European Rapid Alert System for Food and Feed [26].

### 4.1. Detection of STEC in Insects Sold as Feed

Notably, one isolate in our study was identified as a STEC. Shiga toxin-producing *E. coli* are most commonly transmitted via contaminated food, and infections can lead to diseases ranging from diarrhea to severe illness and death [27]. Insects living in farm environments have also been described to function as vectors for STEC, and houseflies were shown to harbor STEC for at least three days after experimental inoculation [28]. However, these insects (two muscid flies and one scarabeid beetle) feed on manure, something that does not happen in the case of the species tested here. *B. mori* exclusively consumes mulberry tree leaves, while locusts favor vegetables, and crickets and tenebrionid beetles are basically omnivorous. However, *Z. atratus* originates from Latin American caves inhabited by fruit-eating bats living in organic wastes and guano [29]. Yet, many beetle generations have passed since then. Still, to the best of our knowledge, no other reports about STEC recovered from edible insects have been published to date. Osimani and colleagues reported the absence of STEC among a variety of edible insect species marketed online [11].

The isolate in our study belonged to serotype O178:H7. O178 STEC have been associated primarily with food of bovine origin in Germany, as well as other countries [30]. Among them, O178:H19 STEC are most commonly reported, but O178:H7 have also been detected in meat and clinical samples from humans [30]. The prototype strain of O178:H7 also carried both *stx1* and *stx2* but lacked *eae* [31]. This strain was also reported to produce enterohaemolysin but, in contrast to our isolate, *ehxA* was not detected. More recently, Miko and colleagues described an O178:H7 variant associated with a *stx1c*/*stx2b*/*ehxA*/*subAB2*/*espI*/[*terE*]/*espP*/*iha* genotype isolated from deer meat and a diseased patient [30]. This is similar to the genotype of our isolate, sharing *stx1c*/*stx2b*/*ehxA*/*subAB*/*iha.* However, *espI*, *espP,* nor *terE* were detected in our study. It is also unclear if the other resistance determinants detected in our STEC isolate might be present in the isolates described by Miko et al., as these were not tested for.

In general, *stx2b* is a variant of *stx2* that is less frequently associated with human disease than the more potent variants *stx2a*, *stx2c*, and *stx2d* [32]. In addition, *eae* is present in the majority of isolates causing severe illness; however, it is not essential for pathogenicity, and *eae*-negative STEC can still cause severe disease, including hemolytic uremic syndrome [27,33]. Between 2012 and 2017, *stx1c*/*stx2b*/*eae*-negative STEC in particular has been associated with 234 human cases in the EU, including at least two cases of HUS, and at least 21 patients required hospitalization [27]. Therefore, a pathogenic potential of the detected STEC isolate cannot be excluded.

Our STEC isolate was obtained from *Z. atratus* sold in a German pet feed store. As these superworms were marketed as feed, they do not fall under the definition of “food” according to Regulation (EU) 178/2002 article 2 a. Besides, samples were raw. Considering the rising interest in edible insects and insect-based foods in the EU and the relative scarcity of currently available products on the market, live insects sold in pet feed stores can seem like an easy, versatile, and appealing alternative for consumers. From a legal point of view, although these products cannot be placed on the market as such, the habit of consuming pet feed insects by themselves seems not explicitly forbidden since Regulation (EU) 178/2002 does not apply to “the domestic preparation, handling or storage of food for private domestic consumption” (Art.1,3). However, the presence of STEC on the tested superworms shows that repurposing insects sold as pet feed to consumers is problematic. Following the recommendations issued for Germany, insects should be cooked for a minimum of 10 min [5]. Although this recommendation is not explicitly valid for pet feed insects, the method is effective to reduce the bacterial loads. Our findings also highlight the fact that appropriate hygiene should be observed by persons purchasing and handling live insects as feed.

### 4.2. Molecular Typing and Antimicrobial Resistance of E. coli from Edible Insect Species

Clustering among the isolates from the pet store according to their PFGE band patterns and cgMLST indicates a comparatively close genetic relationship between the respective isolates. This could be a result of closely related strains circulating in the production facility or within the store selling these animals. The STEC isolate, however, did not cluster with the other isolates.

Predicted serotypes and MLST results were diverse overall. Four isolates belonged to ST10, a very common ST that encompasses isolates from humans as well as animals and clinical as well as commensal strains [34,35]. Sequence types 46 and 101, to which three of our isolates from the Vietnamese silkworm samples belonged, have previously been reported to be very common in neighboring China [36].

In our study, only a single isolate from powdered mealworms was resistant to tetracyclines and carried the gene *tet*(B), which is a common mediator of tetracycline resistance in *E. coli* [37]. In addition, one isolate carried *qnrS2*. Plasmid-mediated quinolone resistance determinants such as *qnr* genes often do not confer clinical resistance to quinolones [38]. However, they can facilitate the development of chromosomal mutations in the quinolone resistance determining regions of the DNA gyrase and topoisomerase genes under treatment [38]. Previous studies have shown that insects such as flies in the farm environment can function as a reservoir and as vectors for the transmission of antimicrobial-resistant bacteria [39,40]. Regarding insects intended for consumption, the few published studies mostly focused on a screening of a certain subset of resistance genes and not on the characterization of individual isolates, including their resistance phenotypes. In three studies conducted in Italy, edible insects (*Tenebrio molitor*, *Locusta migratoria migratorioides,* and samples of a variety of species, respectively) were examined using a very similar method detecting a panel of antimicrobial resistance genes by PCR or nested PCR [41,42,43]. In contrast to our results, all three of these studies reported the presence of various resistance genes. However, the genes examined and detected in these studies are most commonly found in Gram-positive bacteria and are less frequently associated with Gram-negative species such as *E. coli* [37,44]. In a later study by Milanović and colleagues [45], the presence of five genes among the samples of grasshoppers and mealworms was investigated. The authors reported the presence of three different carbapenemase-encoding genes among samples from grasshoppers and mealworms, most notably *bla*_OXA-48_ in over 50% of grasshopper samples. Regarding the clinical importance of carbapenems, these results are concerning. However, the results of these studies do not indicate the bacterial species of the isolates carrying the resistance genes. In contrast to their findings, the susceptibility testing of the *E. coli* isolates in our study showed that all were susceptible to all tested β-lactams.

Overall, it should be noted that several factors influence the microbiome composition and thus likely the resistome as well as the virulence of isolates present in edible insects. This includes the species of insect and the instar, the rearing practices and feed, as well as post-harvest processing, among others [1,46]. Thus, results obtained for samples from different manufacturers, and from different batches produced by the same manufacturer, can vary significantly.

## 5. Conclusions

The detection of a STEC shows that edible insect species, such as *Z. atratus*, can harbor foodborne zoonotic bacteria and highlights the need for hygienic rearing and processing practices to ensure the availability of safe insect-based foods for the interested consumer. On the other hand, the low resistance rate in our study indicates a favorable situation in edible insects in this regard and a low risk of a transmission of antimicrobial-resistant *E. coli*. Considering the high variability of the microbiome of insects, large-scale studies targeting more insect samples as well as different bacterial species are needed to further elucidate potential microbiological risks associated with these foods.

## Figures and Tables

**Table 1:** Antimicrobial resistance phenotypes, antimicrobial resistance genes, and virulence-associated genes of 21 *E. coli* isolates.

Source	Isolate	Resistance Phenotype	Resistance Genotype	Virulence Genes
Insects sold as food	64	-	*mdf(A)*	*gad*, *lpfA*, *terC*
	65	-	*mdf(A)*	*gad*, *terC*, *traT*
	66	-	*mdf(A)*	*gad*, *kpsE*, *kpsMII*, *terC*
	71	-	*mdf(A)*	*gad*, *lpfA*, *terC*
	126	*-*	*qnrS2*, *mdf(A)*	*gad*, *celb*, *terC*
	128	-	*mdf(A)*	*gad*, *astA*, *terC*
	131	-	*mdf(A)*, *sitABC*	*gad*, *sitA*, *terC*, *traT*
	135	-	*mdf(A)*	*gad*, *terC*
	157	-	*mdf(A)*	*gad*, *iss*, *terC*
	160	-	*mdf(A)*	*gad*, *astA*, *celb*, *fyuA*, *irp2*, *terC*, *traT*
	168	-	*mdf(A)*	*gad*, *lpfA*, *terC*
	IP. A	Tetracyclines	*tet*(B), *mdf(A)*	*gad*, *hra*, *terC*
	IP. B	-	*mdf(A)*	*gad*, *lpfA*, *terC*
Insects sold as feed	99	-	*mdf(A)*	*gad*, *hra*, *iss*, *lpfA*, *papC*, *terC*
	100	-	*mdf(A)*	*gad*, *hra*, *lpfA*, *terC*
	101	-	*mdf(A)*	*gad*, *iss*, *terC*
	103	-	*mdf(A)*	*gad*, *hra*, *lpfA*, *terC*
	114	-	*mdf(A)*	*gad*, *cba*, *celb*, *cia*, *cma*, *cvaC*, *ehxA*, *iha*, *ireA*, *iss*, *kpsE*, *lpfA*, *mchF*, *ompT*, *senB*, *stx1A*, *stx1B*, *stx2A*, *stx2B*, *subAB*, *terC*, *traT*
	153	-	*mdf(A)*	*gad*, *iss*, *ompT*, *terC*
	154	-	*mdf(A)*	*gad*, *ccl*, *iss*, *terC*
	156	-	*mdf(A)*	*gad*, *ccl*, *iss*, *terC*

## Data Availability

The data presented in this study are available within the manuscript and the Supplementary Material (Figure S1 and Table S1).

## Supplementary Materials

The following are available online at https://www.mdpi.com/article/10.3390/foods10112552/s1, Figure S1: *Xba*I macrorestriction results including band patterns of individual isolates, Table S1: Assembly statistics and quality parameters of genome sequences.
