# Supplementary material for: Characterization of Escherichia coli from Edible Insect Species: Detection of Shiga Toxin-Producing Isolate
Source: Foods. 2021 Oct 22;10(11):2552. doi: 10.3390/foods10112552 (PMC8618678; doi:10.3390/foods10112552)
Supplement: Supplementary file 1 [file foods-10-02552-s001.zip › foods-1420764-supplementary.pdf]

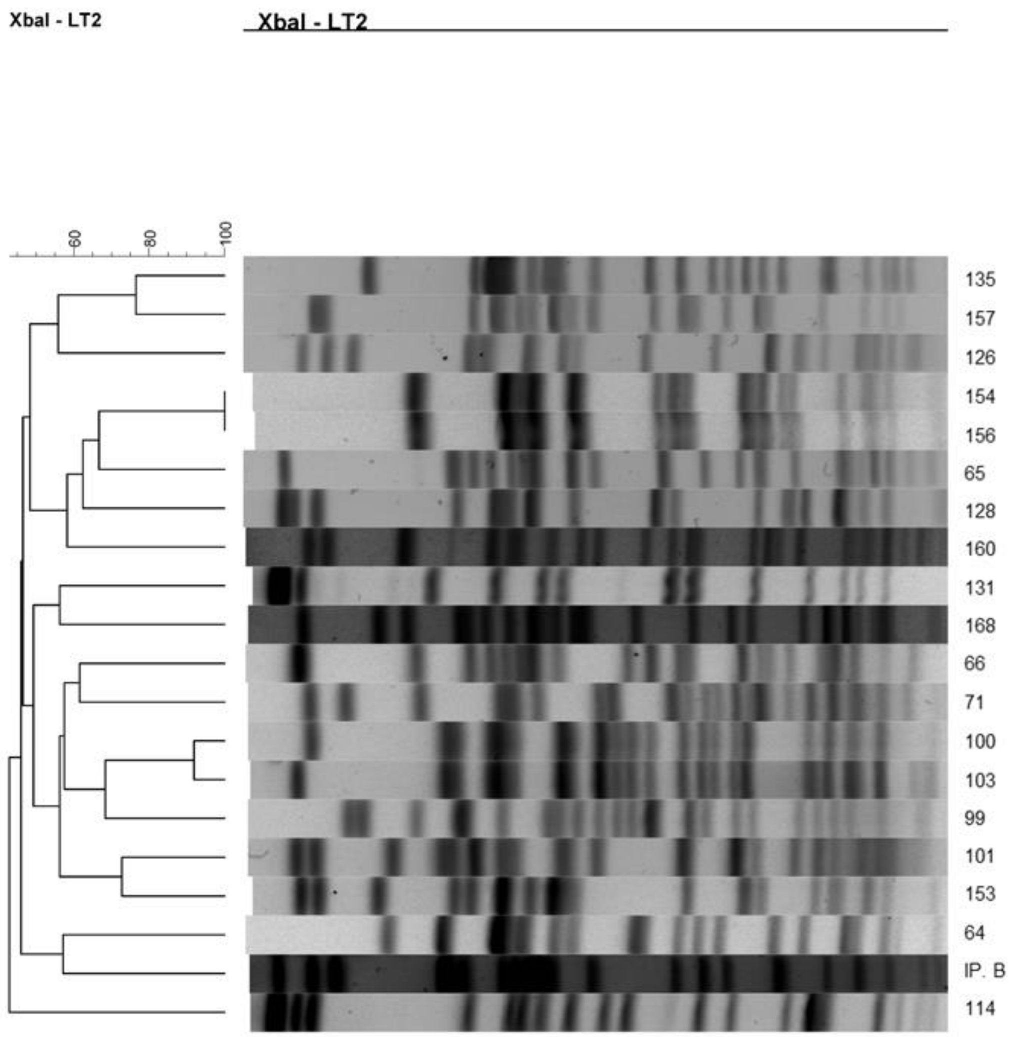

Figure S1. *Xba*I macrorestriction results including band patterns of individual isolates.

Table S1. Assembly statistics and quality parameters of genome sequences.

Isolate ID	# contigs (≥0 bp)	# contigs (≥ 1000 bp)	Total length (≥0 bp)	Total length (≥ 1000 bp)	# contigs	Largest contig	Total length	GC (%)	N50	N75	L50	L75	# N's per 100 kbp
**64**	125	53	4812180	4784632	69	545548	4796453	50.45	212413	119287	8	15	0.00
**65**	156	83	4834403	4811360	91	352142	4817549	50.97	174526	107358	10	19	0.00
**66**	222	74	4928960	4877047	94	391126	4890924	50.61	208998	94308	9	16	0.00
**71**	215	76	5091807	5051299	90	305183	5061310	50.68	150590	82082	13	24	0.00
**99**	159	58	5175455	5141369	71	367270	5151143	50.57	163527	109203	11	20	0.00
**100**	98	38	4869232	4854088	41	761994	4855645	50.84	511187	325760	4	7	0.00
**101**	104	38	4924721	4903580	45	500887	4908911	50.63	213587	137220	8	15	0.00
**103**	105	38	5265049	5246405	42	795016	5248741	50.56	412899	245370	5	9	0.00
**114**	212	94	5337364	5292652	119	651633	5310458	50.59	211470	96659	7	17	0.00
**126**	337	129	4795997	4739800	155	244375	4756419	50.79	90578	45969	17	34	0.00
**128**	141	72	4845249	4822389	81	438216	4828747	50.97	220643	121521	8	15	0.00
**131**	156	79	4856200	4833780	86	367493	4838529	50.60	139646	90875	13	24	0.00
**135**	115	49	4799128	4779429	57	472242	4784459	50.78	207325	121648	8	16	0.00
**153**	118	47	4696194	4673525	56	630882	4679456	50.63	227395	125726	7	14	0.00
**154**	69	35	4713310	4701543	41	640731	4706176	50.77	325377	189889	6	10	0.00
**156**	95	46	4717564	4700929	53	417066	4705837	50.77	210407	111716	8	15	0.00
**157**	78	36	4626437	4614108	38	1178073	4615632	50.76	208379	137285	5	11	0.00
**160**	208	101	5129725	5093085	118	453363	5105314	50.79	137090	61293	12	26	0.00
**168**	237	94	5061773	5016484	114	444072	5030657	50.68	157415	107078	10	20	0.00
**IPA**	163	85	4700400	4675821	96	257563	4683635	50.84	102665	65595	16	31	0.00
**IPB**	89	25	4694634	4673402	35	967268	4680805	50.78	318239	157261	5	10	0.00
